# The voxel-wise analysis of false negative fMRI activation in regions of provoked impaired cerebrovascular reactivity

**DOI:** 10.1371/journal.pone.0215294

**Published:** 2019-05-06

**Authors:** Christiaan Hendrik Bas van Niftrik, Marco Piccirelli, Giovanni Muscas, Martina Sebök, Joseph Arnold Fisher, Oliver Bozinov, Christoph Stippich, Antonios Valavanis, Luca Regli, Jorn Fierstra

**Affiliations:** 1 Department of Neurosurgery, University Hospital Zurich, University of Zurich, Zurich, Switzerland; 2 Clinical Neuroscience Center, University Hospital Zurich, University of Zurich, Zurich, Switzerland; 3 Department of Neuroradiology, University Hospital Zurich, University of Zurich, Zurich, Switzerland; 4 Department of Neurosurgery, Careggi University Hospital, Florence, University of Florence, Florence, Italy; 5 Department of Anesthesiology, University Health Network, University of Toronto, Toronto, ON, Canada; Johns Hopkins School of Medicine, UNITED STATES

## Abstract

Task-evoked Blood-oxygenation-level-dependent (BOLD-fMRI) signal activation is widely used to interrogate eloquence of brain areas. However, data interpretation can be improved, especially in regions with absent BOLD-fMRI signal activation. Absent BOLD-fMRI signal activation may actually represent false-negative activation due to impaired cerebrovascular reactivity (BOLD-CVR) of the vascular bed. The relationship between impaired BOLD-CVR and BOLD-fMRI signal activation may be better studied in healthy subjects where neurovascular coupling is known to be intact. Using a model-based prospective end-tidal carbon dioxide (CO_2_) targeting algorithm, we performed two controlled 3 tesla BOLD-CVR studies on 17 healthy subjects: 1: at the subjects’ individual resting end-tidal CO_2_ baseline. 2: Around +6.0 mmHg CO_2_ above the subjects’ individual resting baseline. Two BOLD-fMRI finger-tapping experiments were performed at similar normo- and hypercapnic levels. Relative BOLD fMRI signal activation and t-values were calculated for BOLD-CVR and BOLD-fMRI data. For each component of the cerebral motor-network (precentral gyrus, postcentral gyrus, supplementary motor area, cerebellum und fronto-operculum), the correlation between BOLD-CVR and BOLD-fMRI signal changes and t-values was investigated. Finally, a voxel-wise quantitative analysis of the impact of BOLD-CVR on BOLD-fMRI was performed. For the motor-network, the linear correlation coefficient between BOLD-CVR and BOLD-fMRI t-values were significant (p<0.01) and in the range 0.33–0.55, similar to the correlations between the CVR and fMRI Δ%signal (p<0.05; range 0.34–0.60). The linear relationship between CVR and fMRI is challenged by our voxel-wise analysis of Δ%signal and t-value change between normo- and hypercapnia. Our main finding is that BOLD fMRI signal activation maps are markedly dampened in the presence of impaired BOLD-CVR and highlights the importance of a complementary BOLD-CVR assessment in addition to a task-evoked BOLD fMRI to identify brain areas at risk for false-negative BOLD-fMRI signal activation.

## Introduction

Task-evoked blood oxygenation-level dependent functional magnetic resonance imaging (BOLD-fMRI) is based on neurovascular coupling and is widely used to interrogate eloquence of brain areas.[1, 2] Neurovascular coupling is considered to represent the biochemical cascade between neuronal activation and subsequent local cerebral blood flow (CBF) increase.[3] This premise, however, imposes two major challenges for an accurate interpretation of BOLD-fMRI studies. First, the extent and strength of the fMRI signal response to a task-related neuronal activation (*for instance finger-tapping*) varies considerably between subjects and therefore requires a summation of repeated tests and arbitrary statistical data thresholding. Second, BOLD-fMRI does not directly measure neuronal activity or blood flow, rather it shows the relative change in de-oxyhemoglobin concentration.[4, 5] The intrinsic BOLD-fMRI signal is therefore strongly influenced by CBF, and to a lesser extent changes in metabolism (cerebral metabolic rate of oxygen (CMRO_2_), and oxygen extraction fraction (OEF)).[2]

Local CBF increases may be limited by the remaining cerebrovasodilatory reserve of the vascular bed.[6] Cerebrovasodilatory reserve can be surmised by measuring BOLD cerebrovascular reactivity (BOLD-CVR) with a sufficient stimulus. [7–10] In case of impaired BOLD-CVR (e.g. a loss of vascular response) the fMRI signal change during task-evoked BOLD-fMRI may not exceed the arbitrary signal-activation statistical threshold[11, 12] despite a functioning neural network, and result in a misleading interpretation of the BOLD-fMRI signal-activation map.[3, 13]

The lack of BOLD-fMRI activation in patients with brain tumors has been termed ‘*neurovascular uncoupling’*, but may actually be a false negative test caused by weakness of BOLD-fMRI signal activation in brain areas adjacent to tumor lesions where BOLD-CVR is impaired.[14, 15] Investigating this aspect, i.e. false negative fMRI activation versus truly absent fMRI signal (*neurovascular uncoupling*), remains challenging in patients.

Therefore, this relationship between impaired BOLD-CVR and BOLD-fMRI signal activation may be better studied in healthy subjects where neurovascular coupling and BOLD-fMRI signal activation are intact. Our laboratory has recently shown the influence of baseline carbon dioxide (CO_2_) on BOLD-CVR and task-evoked BOLD-fMRI measurements.[11] CO_2_ is a potent vasodilator that can be precisely controlled with a prospective model-based end-tidal targeting method (MPET). [8, 16]

For this study, we applied a MPET-controlled normocapnic versus hypercapnic baseline BOLD-CVR study (i.e. normal CVR versus provoked impaired CVR) in order to compare the task-evoked BOLD-fMRI signal responses. By using a voxel-wise analysis, we found that BOLD fMRI signal activation maps are markedly dampened in the presence of impaired BOLD-CVR.

## Materials and methods

We recruited 17 consecutive healthy subjects (age 32±5.5) (ethical institutional approval: Kantonalle Ethikkommission Zürich: KEK-ZH-Nr. 2012–0427). Each subject signed an informed consent form prior to the study and was asked to refrain from heavy exercise, smoking, and caffeine on the day of scanning. The only exclusion criteria was a diagnosed neurological disease.

MRI data were obtained on a 3 Tesla Skyra VD13 MRI (Siemens Healthcare, Erlangen, Germany) with a 32-channel head coil. BOLD parameters used for both BOLD-CVR as well as the task-evoked BOLD-fMRI measurements consisted of axial two dimensional (2D) single-shot EPI sequence planned on the ACPC line plus 20° (on a sagittal image) voxel size 3×3×3 mm^3^, acquisition matrix 64x64x35 ascending interleaved slice acquisition, slice gap 0.3 mm, GRAPPA factor 2 with 32 ref. lines, repetition time (TR)/ echo time (TE) 2000/30 ms, flip angle 85°, bandwidth 2368 Hz/Px, Field of View 192x192 mm^2^. For every subject, we obtained 200 volumes during the CVR study and 135 volumes during the fMRI task-evoked study. A three dimensional (3D) T1-weighted Magnetization Prepared Rapid Acquisition Gradient Echo (MPRAGE) volume was also acquired with the same orientation as the fMRI scans for overlay purposes. Acquisition parameters of the T1-weighted image were: voxel size0.8×0.8×1.0 mm^3^ with a field of view 230x230x176 mm^3^ and scan matrix of 288x288x176, TR/TE/TI 2200/5.14/900 ms, flip angle 8°.

### Controlled CO_2_ stimulus application

End-tidal partial pressure of CO_2_ (PetCO_2_) was controlled and precisely altered by an automated gas delivery system using a computer controlled gas blender with prospective gas targeting algorithms (RespirAct^TM^, Thornhill Research Institute, Toronto, Canada) which allows for automated precise continuous targeting of PetCO_2_ and partial pressure of oxygen (PetO_2_). This method is known as model-based prospective end-tidal targeting (MPET) and described in further detail in work done by Fierstra et al.[8] and Slessarev et al[16]. With MPET, as opposed to other means of controlling PetCO_2_ such as breath holding or inhaling high CO_2_ air mixtures(i.e. 5% or 10% CO_2_), PetCO_2_ is equal to the arterial PCO_2_, the parameter directly influencing brain blood flow.[17] During the entire study protocol, the subject’s blood gases were controlled, independent of breathing frequency or pattern.

### Functional MRI study protocol

**Fig 1:**
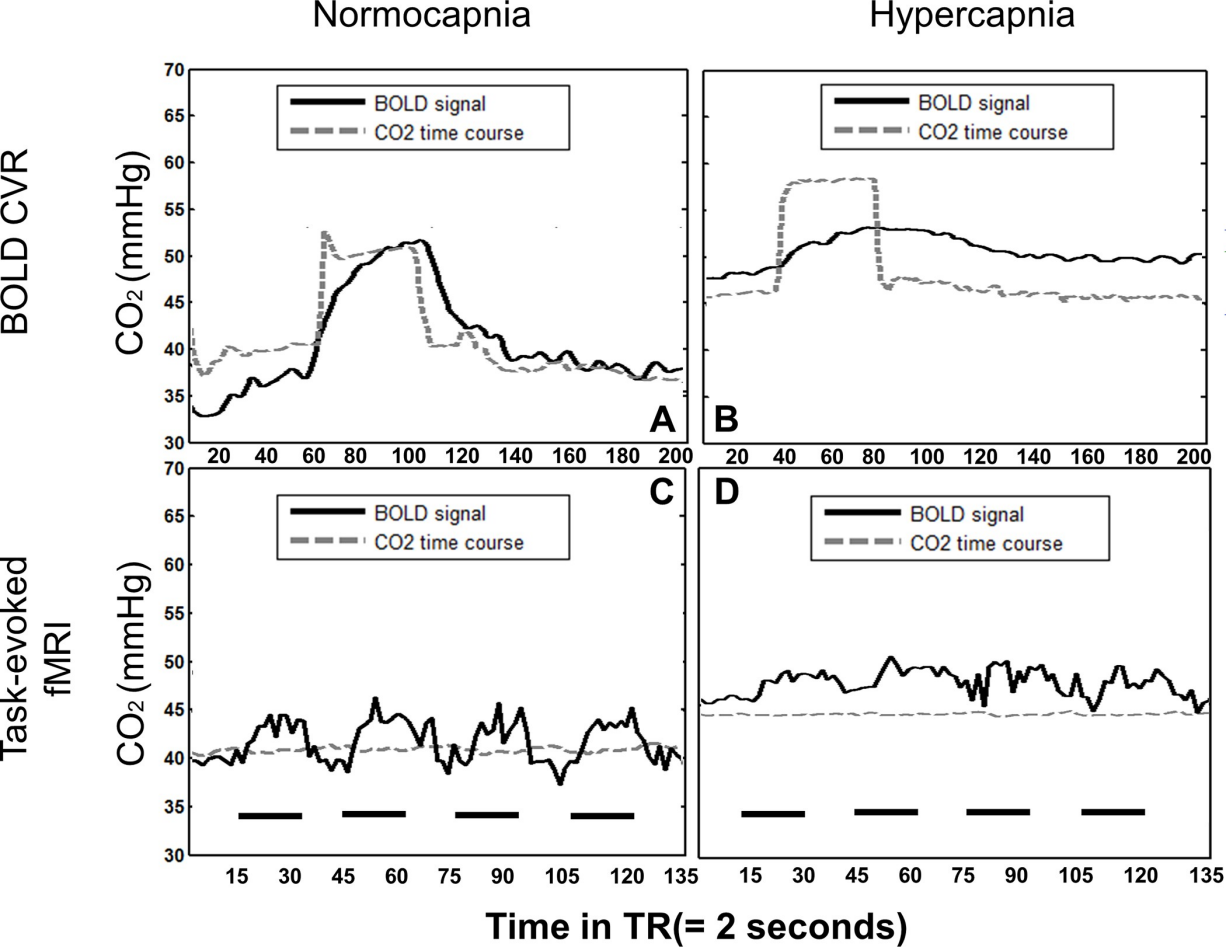
Illustration of the combined BOLD-CVR and task-evoked BOLD-fMRI study protocol in one subject. Top: Fig 1A and 1B show the CO_2_ (grey dotted line) and mean BOLD signal time course (black line) during a BOLD-CVR study at CO_2_ baseline (A) versus hypercapnia (B), respectively. Note that the BOLD signal follows the CO_2_ increase from normocapnia and quickly returns to baseline levels. However, during hypercapnia, the mean BOLD signal increase with CO_2_ rise is less than in normocapnia. Bottom: Fig 1C and 1D show the CO_2_ time course during a task-evoked BOLD-fMRI block protocol. The black short lines represent the task phase of the protocol. The ability of precise CO_2_ control with the MPET method produced a constant CO_2_ level at CO_2_ baseline (C) versus hypercapnia (D), respectively.

Each subject underwent 2 sets of a combined BOLD-CVR scan and a consecutive task evoked BOLD-fMRI scan. During the first BOLD-CVR study, the subjects’ PetCO_2_ was clamped at his/her own resting CO_2_ level for 100 seconds. A pseudo-square wave change of ~ +10 mmHg PetCO_2_ followed and maintained for 80 seconds after which it returned to baseline for several minutes (Fig 1A). The overall duration of one BOLD-CVR data acquisition was 6 minutes and 40 seconds. The BOLD-CVR scan was directly followed by a task-based BOLD-fMRI scan (with a bilateral finger-tapping paradigm). This protocol consisted of 4 blocks of 30 seconds of self-paced bilateral finger-tapping alternating with 30 seconds blocks of rest. Finger-tapping was chosen to enable the visual control of the correct execution of the task. Before starting the BOLD-fMRI scan, the subject’s CO_2_ level was clamped on the same CO_2_ baseline level as for the BOLD-CVR scan (Fig 1C). This scanning protocol, i.e. BOLD-CVR and BOLD-fMRI with bilateral finger-tapping, was then repeated on a higher -hypercapnic- CO_2_ baseline (Fig 1B and 1D). Thus, for both BOLD-CVR and BOLD-fMRI scans, the subjects’ initial CO_2_ baseline was increased and clamped at a hypercapnic level, which was on average ~6 mmHg PetCO_2_ higher than the baseline condition. See Fig 1 to further review the study protocol.

### Image analysis: Spatial pre-processing

Anatomical and functional images were preprocessed using SPM12 (Wellcome Trust Centre for Neuroimaging, Institute of Neurology, University College London, UK). The BOLD images were corrected for slice-timing and realigned to the mean BOLD image. The MPRAGE T1-weighted image was co-registered to the mean BOLD image and probability maps for grey matter, white matter, cerebrospinal fluid, skull, skin and air were generated. The white and grey matter voxels of the functional images were spatially smoothed with an isotropic Gaussian kernel of 6 mm full width at half maximum.

### ROI determination

**Fig 2:**
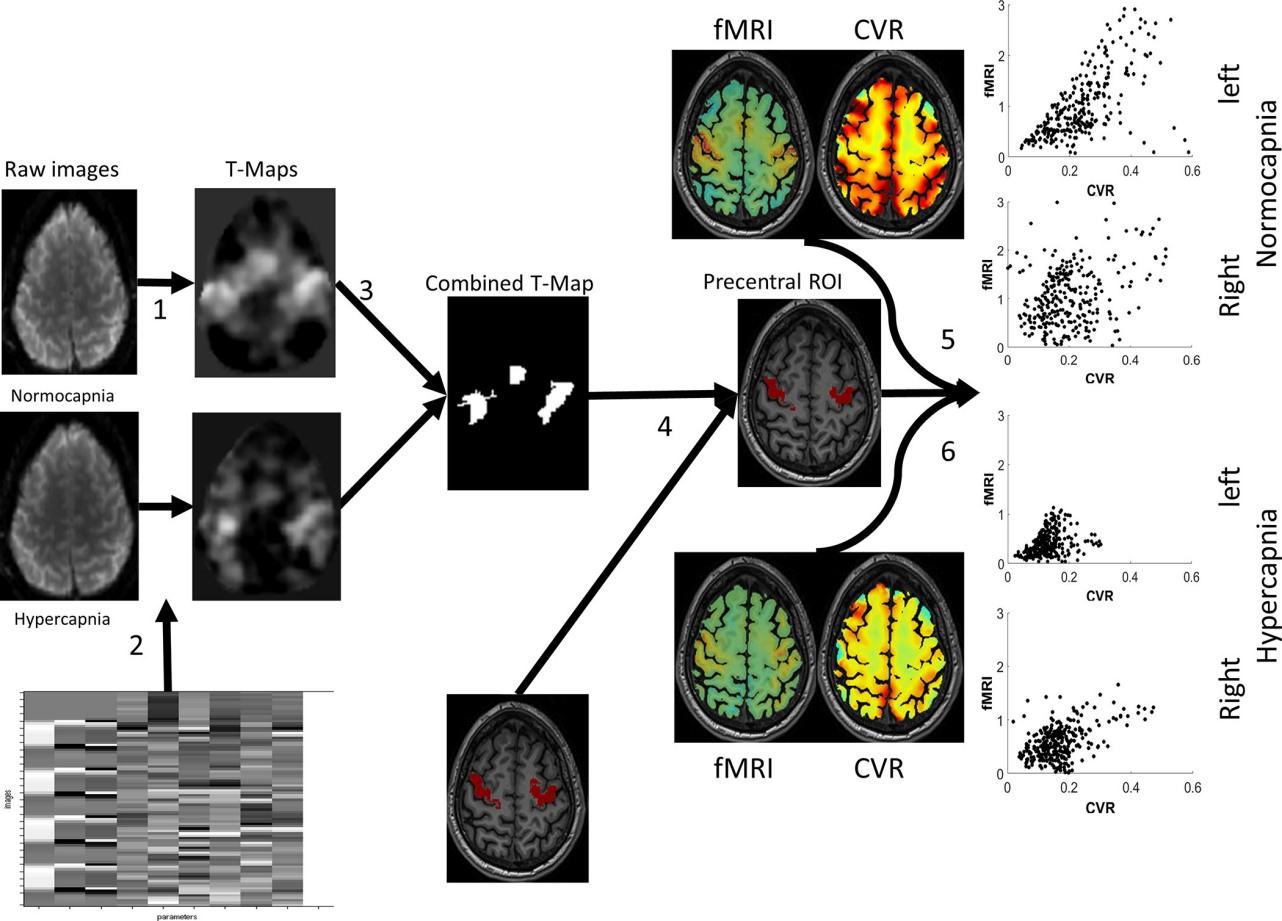
Schematic overview of the analysis pipeline. 1) Representation of the raw BOLD-fMRI data obtained from the MRI system. 2) From the raw BOLD-fMRI images t-value maps are then calculatedusing a mass-univariate general linear model within SPM12. 3) By using a predetermined t-value threshold of 3.43, all significant voxels of each t-value map are combined into a significant-t-value binary mask, i.e. a combined T-map. 4)This map is then overlaid onto the predefined ROIsto determine the significant t-values within each ROI. 5)The BOLD-CVR and fMRI Δ%signal maps for the normocapnic condition are calculated and the values of the combined significant t-value voxels for each region of interest are scatter-plotted. 6)The BOLD-CVR and fMRI Δ%signal maps for the hypercapnic condition are calculated and the values of the combined significant t-value voxels for each region of interest are scatter-plotted. Abbreviations: Blood oxygenation-level dependent cerebrovascular reactivity (BOLD-CVR, defined as %signal change/mmHg CO_2_ change). Functional MRI (fMRI, defined as %signal change).

Anatomical region of Interest (ROI) were derived from the subjects’ specific tissue parcellation using Freesurfer software[18] (http://surfer.nmr.mgh.harvard.edu –see Fig 2). The T1-weighted images were uploaded and automatically segmented in cerebral cortex, subcortical grey matter, lobar white matter, brainstem and cerebellar compartments. From these parcellation maps, we derived different regions associated with the neuronal pathway of finger movements, such as the precentral gyrus, the postcentral gyrus, frontal operculum, and cerebellum. We arbitrarily defined the functional region known as the supplementary motor area (SMA) by combining the paracentral lobule and the frontal superior gyrus.

### BOLD CVR calculations

The BOLD-CVR datasets were further processed with in-house written MATLAB R2016b scripts (The MathWorks Inc., Natick, MA, USA). Temporal smoothing was done by applying a low pass filter and a robust Loess smoothing method to the BOLD-CVR data. CO_2_ time course was interpolated and resampled to match the number of BOLD time points. Secondly, the temporal delay on a voxel-wise basis between CO_2_ and BOLD time course was calculated for improved alignment. BOLD-CVR, defined as percentage BOLD signal change per CO_2_ change in mmHg, was calculated on a voxel-per-voxel basis by regression of the CO_2_ against the BOLD signal time course using a linear least square fitting and determined for the whole brain.[7, 11, 19] BOLD-CVR was color coded and presented as an overlay on the high resolution T1-weighted image, for voxels passing the 0.8 probability threshold in the combined grey and white matter probability map. For each BOLD-CVR data set we also created a t-map as a direct comparison to the BOLD fMRI fingertapping paradigm t-maps.

### BOLD-fMRI processing

After preprocessing, t-values were determined using a first level analysis using a mass-univariate general linear model within SPM12. Head movement correction in 6 directions was included as a covariate to decrease motion artefacts. Voxels were identified as significant if their t-values exceeded the threshold of t-value = 3.43 (average p<0.05 familywise error corrected over all subjects) and a binary mask of significant voxels could then be created (Fig 2). The number of significant voxels for every individual ROI was then evaluated. For all significant voxels of a least one of the two fMRI studies, the mean percent BOLD signal change (Δ%fMRI) was calculated by regression of the block paradigm convolved with the hemodynamic response function with the BOLD signal time course.

For clinical and neuroscientific purposes, we have opted to present both the t-values as well as the % BOLD fMRI signal change throughout the paper.

### Statistical analysis

Statistical analysis was performed using SPSS 23.0 (IBM, Armonk, NY, USA). Continuous data were reported as mean ± standard deviation). The Shapiro-Wilk test was used to assess normality of distribution. Individual differences were determined using a paired student t-test (a p-value of 0.05 was considered significant). Moreover, the mean of all variables between both groups were compared using repeated measured ANOVA to test the variability of the means of each variable between both CO_2_ conditions in the population. For both test, a Bonferroni correction was used to adjust for multiple comparisons. Finally, we constructed a step wise linear regression using a least-squares minimization between the two intra-ROI averaged BOLD-CVR and Δ%fMRI per subject using BOLD-CVR as an independent variable (Fig 2). Statistical significance of the regression was evaluated based on Student t-test, with Bonferroni correction. The data can be found as in the S1 Table as well as accessed through the following website (ftp://www.mr.ethz.ch/hide/marcopi/supplData_CVR_Motor-fMRI_PLOSone2019-2029_EMIDdf17d08d7d0c8542). Request to have access to the raw data files can be made to the corresponding author.

## Results

The data is presented as follows: all BOLD fMRI data (i.e. BOLD-CVR and task based BOLD fMRI signal activation) are given in % BOLD signal change (for BOLD-CVR per mmHg CO_2_ change) as well as in t-values. T-values are given as it represents the more clinical standard for task-based fMRI, while % BOLD signal change is the more commonly used presentation of BOLD-CVR.

### Baseline characteristics

Subject demographics and group averages between both CO_2_ conditions (i.e. baseline versus hypercapnia) can be reviewed in S1 Table. Seventeen subjects (all but one right-handed) were included. None of the subjects had a medical history of neurological intracranial disease, neurological symptoms or was taking medications at the time of scanning. None of the subjects had to be excluded due to excessive head motion. All subjects completed the scanning protocol and no adverse events were reported. Importantly, the CO_2_ increases during the BOLD-CVR study (Fig 1A & 1B) for both protocols was similar (normocapnia: +9.2±0.8 mmHg CO_2_ vs hypercapnia: +9.5±1.4 mmHg CO_2_). Interestingly, the mean BOLD signal during the period of elevated CO_2_ did not differ significantly between both protocols (paired t-test—normocapnia: 344±30 vs hypercapnia: 345±27, p = 0.23). This means that the BOLD signal increase is already saturated with a ~10 mmHg CO_2_ increase and suggests that a CO_2_ step change of ~10 mmHg represents a near maximal stimulus for BOLD imaging. The average BOLD signal in significant activated voxels during the first 30 seconds of the task-evoked fMRI protocol (first 30 seconds of baseline) matched the average BOLD signal during the CVR studies. The average increase in BOLD signal during finger tapping was significantly less than during the BOLD signal step increase during PCO_2_.change. Due to the high variability in the BOLD signal, the ANOVA analysis did not show a significant difference between any fMRI-BOLD signal states.

### Whole brain BOLD-CVR measurements

For each subject, we generated whole brain voxel-per-voxel CVR values after CO_2_ manipulation. (Mean CVR of the whole brain averaged from all scans was 0.19±0.08. The whole brain BOLD-CVR decreased from 0.26±0.05 during normocapnia to 0.12±0.05 during hypercapnia (p<0.001).

### BOLD CVR versus task evoked BOLD-fMRI measurements

Average subjects’ BOLD-CVR and task evoked BOLD-fMRI findings for each anatomical region are listed in Table 1 as well as the mean t-test values of the BOLD-CVR and BOLD-fMRI signal against the CO_2_ evolution.

**Table 1:** Regional CVR and fMRI signal activation and t-values.

		normocapnia		hypercapnia		p-value	
		Δ%signal	t-value	Δ%signal	t-value	Δ%signal	t-value
precentral right	BOLD-CVR	0.22 ± 06	3.6 ± 1.5	0.11 ± 05	0.6 ± 1.0	<0.001	<0.001
	fMRI	0.85 ± 14	5.3 ± 0.8	0.56 ± 22	4.1 ± 1.7	<0.001	0.018
precentral left	BOLD-CVR	0.21 ± 05	3.7 ± 1.6	0.10 ± 05	0.7 ± 1.3	<0.001	<0.001
	fMRI	0.84 ± 16	5.3 ± 0.8	0.53 ± 23	4.0 ± 1.8	<0.001	0.008
postcentral right	BOLD-CVR	0.24 ± 07	3.9 ± 1.7	0.13 ± 06	0.7 ± 1.2	<0.001	<0.001
	fMRI	0.87 ± 17	5.1 ± 1.1	0.60 ± 23	3.9 ± 1.6	<0.001	0.018
postcentral left	BOLD-CVR	0.26 ± 06	3.9 ± 1.6	0.13 ± 05	1.0 ± 1.4	0.003	<0.001
	fMRI	0.87 ± 16	5.3 ± 0.9	0.59 ± 26	4.1 ± 1.8	<0.001	0.019
FO right	BOLD-CVR	0.29 ± 10	4.6 ± 2.1	0.11 ± 04	1.2 ± 1.4	<0.001	<0.001
	fMRI	0.50 ± 23	3.7 ± 1.7	0.29 ± 27	2.1 ± 2.1	<0.001	0.062
FO left	BOLD-CVR	0.34 ± 14	4.5 ± 2.5	0.14 ± 05	1.1 ± 1.5	<0.001	<0.001
	fMRI	0.54 ± 13	3.7 ± 1.6	0.33 ± 23	2.0 ± 1.5	<0.001	0.002
cerebellum right	BOLD-CVR	0.29 ± 08	2.6 ± 1.5	0.16 ± 06	0.6 ± 0.8	<0.001	<0.001
	fMRI	0.70 ± 17	4.5 ± 0.7	0.43 ± 23	2.6 ± 1.3	<0.001	<0.001
cerebellum left	BOLD-CVR	0.28 ± 06	2.7 ± 1.4	0.16 ± 04	0.6 ± 1.0	<0.001	<0.001
	fMRI	0.63 ± 16	4.5 ± 0.7	0.30 ± 22	2.1 ± 1.4	<0.001	<0.001
SMA	BOLD-CVR	0.24 ± 09	3.6 ± 2.1	0.10 ± 04	0.8 ± 1.0	<0.001	<0.001
	fMRI	0.55 ± 16	4.2 ± 0.8	0.41 ± 21	2.5 ± 1.5	0.047	0.004

Abbreviations: BOLD-CVR: CVR values obtained with the blood oxygenation-level dependent fMRI; CVR: cerebrovascular reactivity; fMRI: functional magnetic resonance imaging; FO: frontal operculum; SMA: supplementary motor area

Table 2 depicts the number of activated voxels during each of the CO_2_ conditions. The number of significant voxels, exceeding a t-map threshold of 3.43, decreased significantly for each region during hypercapnia, indicating that false negative fMRI activation can be provoked.

**Table 2:** Number of significantly activated voxels (t>3.43) during task-evoked fMRI.

	normocapnia	hypercapnia	p-value
Precentral right	406 ± 189	280 ± 176	<0.01
Precentral left	454 ± 222	301 ± 171	<0.001
Postcentral right	284 ± 126	172 ± 115	0.009
Postcentral left	352 ± 136	250 ± 122	<0.001
Frontal operculum right	91 ± 109	46 ± 73	0.153
Frontal operculum left	68 ± 82	29 ± 46	0.042
Cerebellum right	388 ± 360	148 ± 174	0.002
Cerebellum left	374 ± 356	110 ± 134	0.001
SMA	1296 ± 1298	569 ± 959	0.001

Values reported in mean ± standard deviation. Abbreviation: SMA: Supplementary motor area

**Fig 3:**
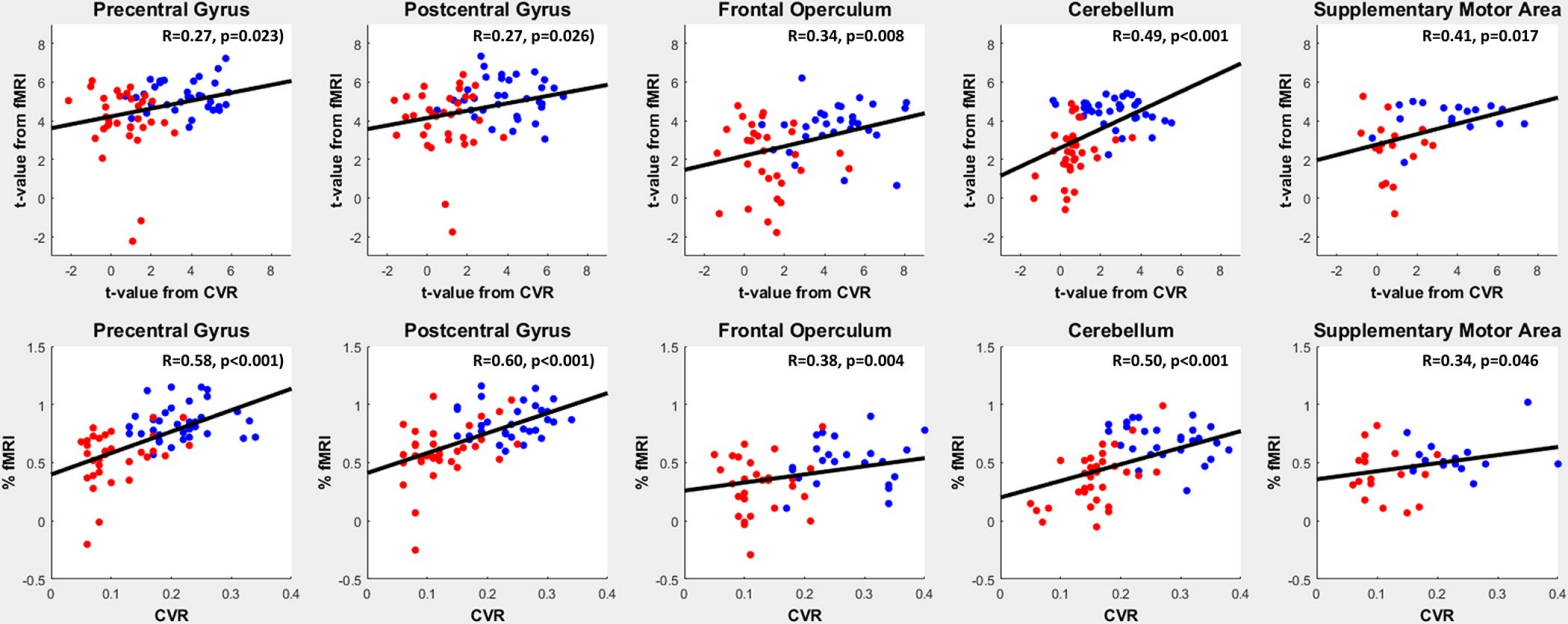
Upper row: Scatterplot of the t-values obtained from the BOLD-CVR and the fMRI finger tapping data. Lower row: Scatterplot of BOLD-CVR % signal change and %fMRI signal change for each ROI and subject. Each red and blue point represents the average value of a ROI of a single subject during either normocpanie (blue) or hypercapnia (red). Black line: least square error linear fit of each scatterplot. Abbreviations: BOLD: blood oxygenation-level dependent; -CVR: cerebrovascular reactivity, CO2: carbon dioxide, %fMRI: mean percent BOLD signal changes, ROI: Region of Interest.

The association between mean BOLD CVR and Δ%BOLD-fMRI was calculated with a linear regression for normocapnia and hypercapnia separately and also for both CO_2_ conditions combined. The statistical results can be found in Table 1 and Fig 3. Fig 3 describes the correlation of the % BOLD signal change, as well as the t-values between BOLD-CVR and task based BOLD fMRI. Combining both CO_2_ conditions resulted in positive correlations between CVR and Δ%fMRI for all anatomical regions.

**Fig 4:**
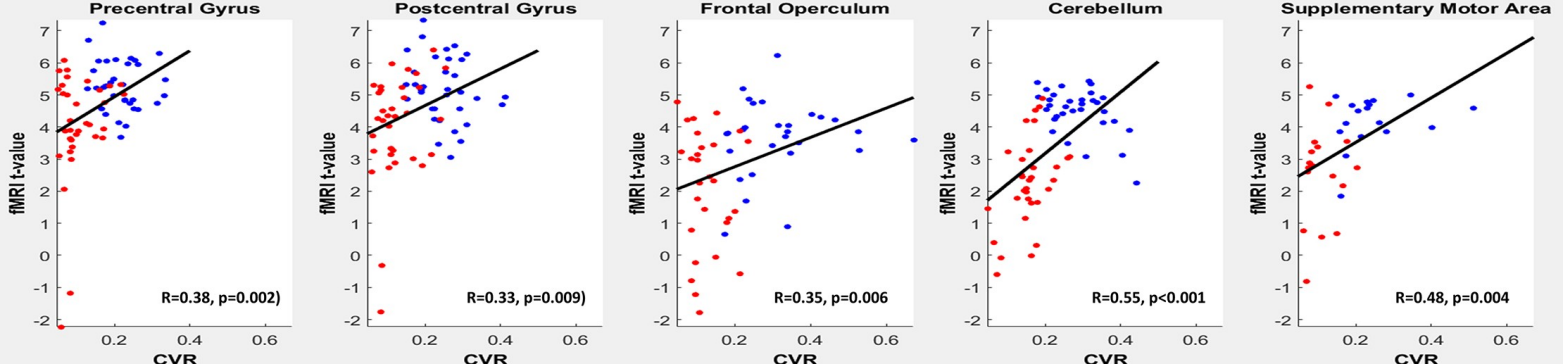
The fMRI t-values are represented as a function of the BOLD-CVR (relative BOLD fMRI signal change per mmHg CO_2_). Each red and blue point represents the average value of a ROI of a single subject during either normocpanie (blue) or hypercapnia (red). Black line: least square error linear fit of each scatterplot. Abbreviations: BOLD: blood oxygenation-level dependent; -CVR: cerebrovascular reactivity, CO_2_: carbon dioxide, %fMRI: mean percent BOLD signal changes, ROI: Region of Interest.

**Fig 5:**
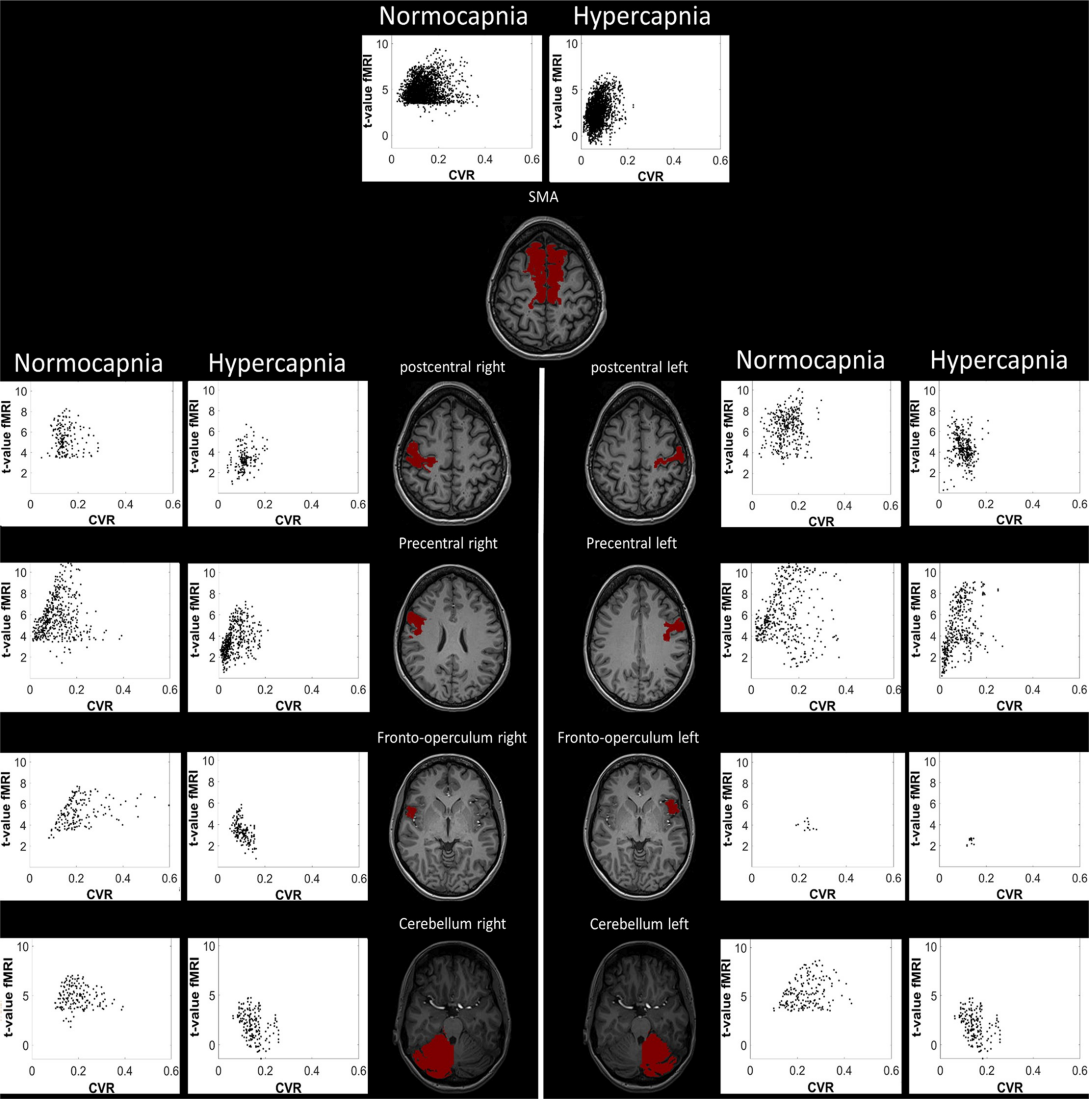
Scatterplot of the voxel-wise t-value of the task-evoked fMRI (y-axis) and BOLD CVR data (x-axis) for an illustrative subject. For each ROI, only the voxels which were significantly activated in at least one of the two finger-tapping fMRI data are plotted. The drop in t-values from normo- to hyper-capnia below the threshold (here 3.43) is clearly visible. This is known as false negative activation, implying the limitation of the task-evoked fMRI by the remaining BOLD-CVR, especially with a fixed threshold.

The correlation between the average BOLD-CVR and fMRI t-values are shown in Fig 4 for clinical relevant interpretation of the data. All correlations are highly significant. Here it is clear that for all anatomical regions during hypercapnia, fMRI t-values drop below the threshold resulting in false negative activation. The voxel-by-voxel Δ%fMRI vs. CVR response and corresponding t-value comparison of an illustrative subject during both CO_2_ conditions is shown in Fig 5. Interestingly, in every ROI, t-values of fMRI are strongly related to BOLD-CVR. The t-values does not seem to surpass a certain CVR threshold, indicated by the upper distribution of the voxels presented in the graph. Past this threshold, t-values shows a strong association with BOLD-CVR until around a CVR between 0.2 and 0.3 where the t-values does not seem to increase further. This threshold seems to differ only slightly between subjects. Such a pattern is not only seen in Fig 5, but can also be seen in the scatter plots in Fig 2.

In some voxels, the t-values in response to a fingertapping paradigm are clearly not maximal. Many voxels, which still significantly participate in the neuronal pathway show lessened fMRI activation even with high CVR. To localize these voxels with low fMRI signal activation but a preserved CVR, we applied a Δ%fMRI threshold of <1 and a CVR threshold of >0.15 to the activated voxels in the precentral and postcentral gyrus. As expected, the voxels with less activation are located to a greater extent in the periphery of the sensorimotor cortex and cortical areas with significantly higher activation. As the vasodilatory capacity decrease during hypercapnia, fMRI t-values, especially in primary activated regions like the pre- and postcentral gyrus, are increasingly correlated with BOLD-CVR.

## Discussion

The main finding of this study is that false negative BOLD fMRI signal activation occurs when BOLD-CVR impairment is simulated in healthy subjects. This findings could be consistently reproduced in all participants. The dampened BOLD fMRI signal response reveals yet another major uncertainty of accurate BOLD-fMRI data interpretation. Quantitative voxel-wise analysis of t-values and Δ% signal of BOLD-CVR and fMRI effects were strongly correlated over the whole cerebral motor network, accounting for more than half of the variance. This highlights the importance of the complementary role of BOLD-CVR to identify brain areas at high risk for false negative BOLD fMRI signal activation, in particular for clinical interpretation of fMRI data, e.g. pre-operative planning of brain tumor surgery in eloquent areas. Here it is known that by using low fMRI t-value thresholds more noise is increasingly introduced into the fMRI activation maps, thereby limiting the interpretability of the maps. When in addition the BOLD-CVR is also reduced in these eloquent regions, the fMRI study can be considered insufficient for clinical interpretation. Prior to BOLD-CVR studies in brain tumor patients, this absent fMRI signal activation was considered as a result of adaptive brain plasticity (i.e. lateralization of fMRI signal to the contralateral hemisphere). However, a more feasible explanation is that in the presence of impaired BOLD-CVR, fMRI-“silent” or -“invisible” activation maps may occur because of an absent or at best a minimal BOLD response to an appropriate neural stimulus.[13, 20] Additionally, local mass effect from brain tumors can also have a significant effect on extend of CVR impairment.[15] This may also be true for vascular lesions such as arteriovenous malformations, where arterial-venous shunting can induce a similar effect on BOLD-CVR. Here false negative activation is more likely to occur near such a lesion, potentially increasing the risk of adverse surgical outcomes.[13, 21]

During protocol 2 (i.e. hypercapnic baseline) BOLD-CVR (Δ%Signal and t-values) could be attenuated in all subjects. This attenuation correlated significantly to a decreased task-evoked BOLD-fMRI (Δ%Signal and t-values) in voxels previously active during normocapnia (i.e. protocol 1). We interpret this as false negative fMRI signal activation. Neurons in these areas are known to be viable but are unable to produce a sufficient vascular response to exceed the arbitrarily set (but commonly used) signal activation threshold.

The inability to differentiate false negative activation from neurovascular uncoupling has been identified by others who have suspected an induced false negative activation. However, this is the first report of combined BOLD-CVR and task-evoked fMRI changes during different same-session controlled CO_2_ conditions. For instance, Halani et al. reported only BOLD-CVR during different CO_2_ conditions and showed a decreased BOLD-CVR response during hypercapnia.[22] Moreover, Cohen et al. only studied the task evoked BOLD fMRI response and showed a decreased signal activation after acetazolamide application.[23] Whittaker et al showed that the relative CBF response difference of a neuronal task during uncontrolled hypercapnia (~+3-4mmHg) was reduced, but not the BOLD fMRI signal response.[24] This finding of a preserved BOLD fMRI signal response is potentially due to the mild CO_2_ increase. In our study, we provided a subject-wise quantitative comparison between both BOLD-CVR & BOLD fMRI measurements using a uniform controlled CO_2_ stimulus which allowed us to reproduce and expand upon the knowledge gained from previous studies. Moreover, we have analyzed BOLD fMRI measurements for multiple anatomical regions in the brain, known to be activated during a bilateral finger-tapping paradigm, to obtain a comprehensive overview of BOLD fMRI responses. We report that the BOLD-CVR and BOLD-fMRI activations for each ROI exhibit a similar and specific pattern for every subject (Figs 2 and 5).

### Provoking reduced BOLD-fMRI activations

We only included young healthy subjects without known neurological disease or symptoms in order to expect intact cerebrovascular autoregulation and neurovascular coupling during baseline conditions. In a recent study, we have shown the importance of subjects’ resting CO_2_ state for accurate BOLD-CVR and BOLD fMRI measurements.[11] The current work elaborates on these findings and extends them to the relationship between BOLD-CVR and false negative activation in known viable neurons. Here, in response to hypercapnia-induced reduction in vasodilatory capacity, the BOLD-fMRI t-values drop below the task BOLD-fMRI t-value significance threshold, which may be interpreted as negative activation. With the knowledge that these areas did respond adequately during normocapnia, such negative activation can be seen as *false* negative activation (i.e. type II error). When BOLD-CVR values remain within the normal range (here >0.2% BOLD signal Change/mmHg CO_2_), such false negative activation is unlikely to occur. Conversely, if BOLD-CVR is significantly impaired, alternate investigations to identify eloquent regions are warranted. It appears prudent that a standardized BOLD-CVR study accompany all task-evoked fMRI studies, particularly in patients at risk of false negative activation, i.e. patients with brain tumors in any location.[15, 25] Our study showed a decrease in the number of significant voxels in all anatomical regions of the brain (see Fig 5).

For task-evoked BOLD-fMRI studies, the traditional assumption remains that neuronal activation does not induce a maximal fMRI signal response. Based on a comprehensive evaluation of the motor network ROIs, we now demonstrate that BOLD fMRI increases correlate with the remaining vasodilatory capacity (i.e. BOLD-CVR). However, our data suggests that this correlation is due to a BOLD-fMRI activation saturation effect for every anatomical region, as shown on ROI averaged data (Figs 3 and 4) or on voxel-wise data (Fig 5)

### Reduced deoxyhemoglobin washout

We showed that compared to a normocapnic baseline, there was an average ~50% reduction in BOLD-CVR measured during the hypercapnic baseline protocols (BOLD-CVR for normocapnia: 0.26 versus hypercapnia: 0.12 Δ%signal/mmHg CO_2_). This ~50% reduction was very consistent, and found for all analyzed individual anatomical regions. Work by others has shown smaller BOLD-CVR reductions of about 10–30% with a preset hypercapnic baseline.[12, 22, 24] This large difference in BOLD-CVR reduction may be explained by the use in those studies, of a preset CO_2_ baseline not representative of the subjects’ own physiological baseline. Additionally, in these studies the fMRI signal was inferred from the control images of a pulsed Arterial Spin Labeling sequence. Theoretically, it may be very favorable to obtain fMRI and CBF measurements simultaneously, however, such combined BOLD-ASL sequences result in suboptimal fMRI measurements with a significant reduction in spatial and temporal resolution.[26] This limitation is often ignored in studies reporting such data. The difference in resolution can be seen in the average BOLD-CVR values of ~0.18% signal change/mmHg CO_2_, which is ca. 30% lower compared to our findings in healthy subjects and findings by others using BOLD sequences exclusively.[27, 28]

The use of a simultaneous BOLD-ASL sequence is a result of an ongoing discussion suggesting that BOLD-fMRI signal changes do not represent CBF changes directly, but rather represent an unknown interaction between different hemodynamic factors (e.g. cerebral blood volume, oxygen extraction fraction, and cerebral metabolic rate of oxygen).[2, 29] Two papers directly comparing BOLD-CVR with both ASL and (^15^O)-H_2_O Positron Emission Tomography derived CBF perfusion reserve in patients with cerebrovascular steno-occlusive disease, show a good agreement.[7, 30] Findings by Halani et al. elaborated on these findings in healthy subjects.[22] They confirmed this association between BOLD-CVR and CBF measurements, but also demonstrated that the strength of the association is dependent on the resting vasodilatory state before a vasoactive stimulus is applied. This finding reaffirms the importance of the influence of baseline CO_2_, which influences the vascular tone[11], on BOLD-CVR measurements.

### Limitations

This model we investigated is based on a group of younger healthy subjects. Eminent is the validation of this model in older healthy subjects as well as in different patient cohorts (i.e. patients with steno-occlusive disease and patients with gliomas). Validation is necessary to test the agreement between our model and pathophysiological reduction in CVR on fMRI signal activation. Therefore there is a need for implementation of a BOLD-CVR study as a corroborating study to fMRI.

In this study we have opted for an arbitrary fMRI t-map threshold to include only significant voxels. We used a t-value of 3.43, which is the average p<0.05 familywise error corrected over all subjects. Probably, a higher t-value threshold would have resulted in a stronger association between both measurements. However, we believe that the threshold, chosen by us, is a good representative of activated voxels, especially in potential future clinical settings. This can also be seen in Fig 2, where voxels bordering those high activated voxels are still taken into account.

## Conclusion

In healthy subjects, false negative BOLD fMRI signal activation can be found when cerebrovascular reactivity is artificially impaired. Our findings highlight the importance of the complementary role of BOLD-CVR to identify brain areas at high risk for false negative BOLD fMRI signal activation.

## Supporting information

S1 Table Final dataset Plos ONE. (XLSX)
